# The journey back to normality: Support systems and posttrauma needs following exposure to single‐incident trauma among children and adolescents

**DOI:** 10.1002/jts.22902

**Published:** 2023-01-02

**Authors:** Katharina Haag, Rachel Hiller, Rosie McGuire, Mark Lyttle, Sarah L. Halligan

**Affiliations:** ^1^ Department of Psychology University of Bath Bath United Kingdom; ^2^ Emergency Department Bristol Royal Hospital for Children Bristol United Kingdom; ^3^ Research in Emergency Care Avon Collaborative Hub University of the West of England Bristol United Kingdom; ^4^ Department of Psychiatry and Mental Health University of Cape Town Cape Town South Africa

## Abstract

Social support has been linked to posttrauma adjustment in children and adolescents, but the components of good support remain poorly defined. We conducted qualitative interviews with 30 youths aged 7–16 years after being admitted to a hospital following a single‐incident trauma, predominantly injury or illness. The aim was to identify youths’ support needs and examine the support they received across different recovery stages. Thematic analysis revealed that although participants appreciated increased attention and warm support during their hospital stay, most wanted their lives to return to normal soon afterward and were frustrated by barriers to achieving this. Participants received support from different sources, but parents and peers were the most important providers of emotional support and the people with whom these individuals most frequently engaged in trauma‐related conversations. Furthermore, although it was important that schools were sensitive to the youths’ potential limitations regarding their ability to engage with lessons, emotional support from teachers was less valued. Overall, this study implies that ecological models incorporating multiple interacting layers capture the structure of youths’ posttrauma support systems well. These findings may be used to tailor posttrauma interventions more closely to child and adolescent needs at different recovery stages and highlight the importance of having parents and, where possible, peers involved in posttrauma interventions or prevention programs.

## 抽象

Traditional and Simplified Chinese Abstracts by the Asian Society for Traumatic Stress Studies (AsianSTSS)

簡體及繁體中文撮要由亞洲創傷心理研究學會翻譯

Traditional Chinese

恢復正常的旅程 ‐ 年輕人在遭受單一事件創傷後的支持系統和需求

摘要

社會支持與兒童和青少年的創傷後適應有關, 但良好支持的組成部分仍然不明確。我們對30名7‐16歲的青年進行了定性訪談, 他們因單一事件創傷(主要是受傷或疾病)而入院。目的是確定青少年的支持需求, 並研究他們在不同的康復階段所獲得的支持。專題分析顯示, 儘管參與者對住院期間增加的關注和溫暖的支持表示感激, 但大多數人希望他們的生活在住院後很快恢復正常, 並對實現這一目標的障礙感到沮喪。參與者從不同的來源獲得支持, 但父母和同齡人是最重要的情感支持提供者, 也是他們最常與之進行創傷相關對話的人。另外, 儘管學校對青少年參與課程能力的潛在局限性有敏感認識, 但教師的情感支持卻不那麼受重視。總的來說, 本研究表明, 生態模型包含多個相互作用的層次, 很好地捕捉了青少年創傷後支持系統的結構。這些發現可用於更貼近兒童和青少年在不同恢復階段的需求的創傷後乾預措施, 並強調讓父母和在可能的情況下, 同齡人參與創傷後乾預或預防計劃的重要性。

恢复正常的旅程 ‐ 年轻人在遭受单一事件创伤后的支持系统和需求

社会支持与儿童和青少年的创伤后适应有关, 但良好支持的组成部分仍然不明确。我们对30名7‐16岁的青年进行了定性访谈, 他们因单一事件创伤(主要是受伤或疾病)而入院。目的是确定青少年的支持需求, 并研究他们在不同的康复阶段所获得的支持。专题分析显示, 尽管参与者对住院期间增加的关注和温暖的支持表示感激, 但大多数人希望他们的生活在住院后很快恢复正常, 并对实现这一目标的障碍感到沮丧。参与者从不同的来源获得支持, 但父母和同龄人是最重要的情感支持提供者, 也是他们最常与之进行创伤相关对话的人。另外, 尽管学校对青少年参与课程能力的潜在局限性有敏感认识, 但教师的情感支持却不那么受重视。总的来说, 本研究表明, 生态模型包含多个相互作用的层次, 很好地捕捉了青少年创伤后支持系统的结构。这些发现可用于更贴近儿童和青少年在不同撤消阶段的需求的创伤后干预措施, 并强调让父母和在可能的情况下, 同龄人参与创伤后干预或预防计划的重要性。

## Resumen

JOTS‐22‐0355.R2 sp/abs

Spanish Abstracts by Asociación Chilena de Estrés Traumático (ACET)

El Viaje de Regreso a la Normalidad: Sistemas de Apoyo a los Jóvenes y necesidades después de la exposición a trauma de incidente único

NECESIDADES POST‐PANDEMIA Y APOYO EN LA JUVENTUD

El apoyo social se ha relacionado con el ajuste postraumático en niños y adolescentes, pero los componentes de un buen apoyo permanecen poco definidos. Realizamos entrevistas cualitativas con 30 jóvenes de 7 a 16 años de edad después de ser admitidos en un hospital luego de un trauma de incidente único, predominantemente una lesión o enfermedad. El objetivo era identificar las necesidades de apoyo de los jóvenes y examinar el apoyo que recibieron en las diferentes etapas de recuperación. El análisis temático reveló que, aunque los participantes apreciaron una mayor atención y un cálido apoyo durante su estadía en el hospital, la mayoría deseaba que sus vidas volvieran a la normalidad poco después y se sentían frustrados por las barreras para lograrlo. Los participantes recibieron apoyo de diferentes fuentes, pero los padres y los compañeros fueron los proveedores más importantes de apoyo emocional y las personas con quienes entablaron conversaciones relacionadas con el trauma con mayor frecuencia. Además, aunque era importante que las escuelas fueran sensibles a las limitaciones potenciales de los jóvenes con respecto a su capacidad para participar en las lecciones, el apoyo emocional de los maestros fue menos valorado. En general, este estudio implica que los modelos ecológicos que incorporan múltiples capas interactivas capturan bien la estructura de los sistemas de apoyo postraumático de los jóvenes. Estos hallazgos pueden usarse para adaptar las intervenciones postraumáticas más de cerca a las necesidades de los niños y adolescentes en diferentes etapas de recuperación y resaltar la importancia de tener padres y, cuando sea posible, compañeros involucrados en las intervenciones postraumáticas o programas de prevención.

Hospital admission following serious injury or illness is common in children and adolescents (Watson & Errington, 2016). The recovery process can be lengthy, comprising practical (e.g., hospitalization, medical procedures) and psychological (e.g., emotional distress, disruption of routines) challenges. This can put significant pressure on youths, (i.e., individuals under 18 years of age) and their families (Ramsdell et al., 2015). As a result, approximately 11% of youths show persistent symptoms of posttraumatic stress disorder (PTSD) up to 1 year after exposure to a potentially traumatic event (PTE; Hiller et al., 2016). As only a subset of youth exposed to PTEs develop persistent problems (Alisic et al., 2014), it is important to better understand which factors support adaptive outcomes and what challenges youth may face on their way to recovery.

Previous work has established the importance of social support as a predictor of posttraumatic stress symptoms (PTSS) among children and adolescents (Hiller et al., 2018; Trickey et al., 2012). Qualitative studies show that most parents adopt a responsive parenting style, characterized by recognizing and acting upon their child's needs (Alisic et al., 2011b), with a focus on preventing future harm (Williamson et al., 2016). These parents engage in warm and sensitive behaviors, offer reassurance, aim to keep up routines, and provide opportunities to discuss the traumatic experience (e.g., Alisic et al., 2011b; McGuire et al., 2019). However, some parents describe struggling with supporting their children posttrauma, engaging in potentially maladaptive strategies, such as encouraging avoidance coping to minimize their child's immediate distress (e.g., Williamson et al., 2016, 2019). Although this is an understandable response, longitudinal research has shown that avoidance can maintain children's PTSS (Hiller et al., 2018). Overall, these studies provide a good picture of parental efforts to support their children but offer limited insight into what optimal support may look like from a child or adolescent's perspective.

Studies on other sources of social support are scarcer. Although trauma‐informed care by first responders and emergency department (ED) and hospital staff may positively influence outcomes (Marsac et al., 2019), perceptions of problematic ED care (e.g., a lack of information, disruption of parental efforts to ease children into posttrauma conversations) can increase child and parental distress (Alisic et al., 2012; Williamson et al., 2016). Peer support becomes more important beginning in middle childhood (e.g., Alisic et al., 2011a) and has been linked to lower PTSS in youth (e.g., Morley & Kohrt, 2013). At school, teachers are frequent points of contact, but they may vary with regard to their confidence and competence in providing support depending on their level of knowledge or direct experience with trauma (e.g., Alisic et al., 2012). The wider school context is understudied, although a lack of a school's willingness to flexibly adapt to youths’ needs has been found to lead to strained relationships with families (Røkholt et al., 2016).

In summary, youth commonly receive support from multiple sources following trauma exposure, reflecting an ecological conceptualization with multiple interacting layers (van Wesel et al., 2012). However, few studies have examined the complexity of these support structures in‐depth, and very few have interviewed children and adolescents to understand their perspectives on what constitutes ideal support from each source. In one study, a sample of 8–12‐year‐olds who had experienced a single‐incident trauma discussed their appreciation of tangible signs of support from family members and friends, including gifts, visits, sympathy, and practical help (Alisic et al., 2011a). However, the information provided about different types of support from different actors (e.g., parents, peers, siblings) was limited. Other studies have typically focused on individual‐level coping processes rather than support structures (e.g., Salter & Stallard, 2004). A more detailed examination of how the wider social context can meet youths’ support needs following trauma is warranted, particularly during the first 6 months, which are central to recovery (Hiller et al., 2016). To address this gap, we conducted interviews with 30 youths aged 7–16 years between 1 and 6 months after they had been hospitalized due to injury, illness, or assault. We investigated support from various sources and examined changes over time, with the goal of providing a more nuanced picture of youths’ complex posttrauma social support systems and needs.

## METHOD

### Participants

Participants were 30 children and adolescents (age range: 7–16 years) and one of their parents. Using a purposive sampling approach, families were recruited following child ED attendance after exposure to a traumatic event, predominantly accidental injury. All traumatic events qualified for PTSD Criterion A per the *Diagnostic and Statistical Manual of Mental Disorders* (5th ed.; *DSM‐5*; American Psychiatric Association, 2013). Participants were assessed 1–6 months following their initial ED admission (*M* = 92.47 days, *SD* = 46.15, *Mdn* = 87.50 days), capturing experiences at varying recovery stages. Exclusion criteria were insufficient fluency in English, suspicion of deliberate self‐harm or suicidal intent, intellectual disability precluding mainstream schooling, history of organic brain damage, and/or child protection involvement or suspicion of carer neglect or abuse. A total of 54 families were approached, 30 (53.6%) of whom participated. Reasons for nonparticipation were the research team's inability to contact the parents (*n* = 14), parents declining to participate (*n* = 6), participants dropping out after having been booked into the study (*n* = 2), and family‐specific reasons (*n* = 2). There were no significant differences between the recruited versus not recruited groups for child age, *t*(54) = .95; *p* = .346; sex, χ^2^(1, *N* = 57) = 2.85, *p* = .091; number of injuries, *t*(46) = .90, *p* = .374; and whether the child was seen in the resuscitation department, χ^2^ (1, *N* = 55) = 1.40, *p* = .237. Groups differed significantly with regard to triage category, χ^2^(3, *N* = 47) = 8.55, *p* = .036, with 85.7 % (*n* = 18) of participants showing a triage rating of 1 (i.e., “requires immediate attention”) compared to 46.2 % (*n* = 12) of nonparticipants.

The mean participant age was 12.0 years (*SD* = 2.55), and 76.7% were male. Seven individuals reported symptom levels indicating probable PTSD, and another three reported mild‐to‐moderate PTSD symptoms. The average Child and Adolescent Trauma Screen (CATS; Sachser et al., 2017) score for PTSS symptoms was 12.79 (*SD* = 10.13, range: 1–39; see Table 1 for further descriptive information).

**TABLE 1:** Child and parent descriptive information

**Variable**	***M***	***SD***	**Range**	***n***	**%**
***Child characteristics***					
Age (years)	12.03	2.55	7‐16		
Male sex				23	76.7
Caucasian race/ethnicity				30	100.0
Trauma type					
Motor vehicle accidents (pedestrian or in car)				9	30.0
Other severe accidents/injuries (e.g., sports injuries, falls, amputations, cycling accidents)				18	60.0
Acute life‐threatening illness				2	7.0
Assault				1	3.0
Triage category^a^					
Immediate attention required				18	90.0
Very urgent				1	0.5
Urgent				2	1.0
Less urgent				0	0.0
Days in hospital	6.37	7.28	1–35		
Days of missed school	18.45	25.06	2–120		
Details of traumatic event					
Ambulance/helicopter required				23	76.6%
Bone fracture				12	40.0
Head injury				14	46.6
***Parent characteristics***					
Age (years)	42.60	5.22	31‐50		
Patient's mother				24	80.0%
Married or cohabiting				21	70.0
Educational attainment					
School until 18 years of age or younger				6	20.0
Further education (vocational training/diplomas)				10	33.3%
Higher education				14	46.6
Pretax household income (GBP)					
< ₤40,000				7	23.3
₤40,000–₤100,000				13	43.3
> ₤100,000				4	13.3
Not sure/parent did not wish to respond				6	20.1

*Note*. ^a^Data missing for nine participants; percentages are provided based on the whole sample.

### Procedure

Research nurses identified potentially eligible families who presented at the ED of a large children's hospital and asked their permission to be contacted by the researchers either telephonically or in person. Upon being recruited into the study, a researcher visited each family at their home between March 2017 and November 2019; visits lasted 60–90 min. To allow participants to warm up and get to know the researchers, 5–15 min were spent talking about personal interests and everyday life. Informed consent or assent was then obtained from parents and youth, respectively. Families were informed about the study's purpose and why the researchers felt it was important, but researchers highlighted that individuals could refuse participation or withdraw at any time without any negative consequences. Next, the youth participants filled in the CATS, and their parents completed the demographic questionnaire. Then, the youth completed a timeline task, which served as the foundation for semistructured interviews. Researchers noted key takeaways and any relevant information for interpreting the interview (e.g., child nervousness) after its completion. A standard risk protocol was in place, but no risk events were identified. Ethical approval was obtained from the University of Bath Psychology Research Ethics Committee and the National Health Services (NHS) Health Research Authority South West–Central Bristol Research Ethics Committee.

### Measures

#### Demographic characteristics

Parents completed a demographic questionnaire, providing details about the child and family, the child's psychiatric history, the traumatic event that necessitated ED attendance, and the youths’ support needs in the time since the ED visit.

#### PTSS

The CATS (Sachser et al., 2017) was used to assess youth PTSS. Participants reported the presence of 20 *DSM‐5* symptoms over the 2 weeks prior, rating responses on a scale of 0 (*never*) to 3 (*almost always*). Based on the established cutoffs for this measure, symptom scores of 0–14 were categorized as nonelevated symptoms, 15–20 as mild‐to‐moderate PTSS, and 21 or more as probable PTSD. The CATS has demonstrated good internal consistency and discriminant validity (Sachser et al., 2017). In the present sample, Chronbach's alpha was .88.

#### Youth needs and social support

Two members of the research team, both female PhD students trained by experienced qualitative researchers, conducted the home interviews. The participants chose whether they wanted their parents to stay in the room or leave to give them privacy, with 21 choosing the former option. Present parents were asked to only listen and not participate in the interviews or provide answers on behalf of their children. Nonetheless, several parents spontaneously intervened to encourage honest answers or elaboration, potentially facilitating disclosure.

An interview guide was developed in cooperation with researchers experienced in interviewing children and aimed to assess participants’ support needs and their main sources of social support at different recovery stages following hospitalization. After the first three interviews were conducted, small revisions in phrasing and content were made, with input from a trained clinician, to make the interviews more accessible for younger and shy children. At the start of the interview, to facilitate discussion, participants were presented with a picture‐based timeline covering six discrete time points: before the PTE, during the PTE, at the hospital, when returning home, when returning to school, and the present. Participants were given several pictures of emoji faces with a descriptive word written below and asked to indicate how they felt at each time point. They could use multiple emojis to describe their feelings at one given time and add emotions by writing them onto additional cards. The information from this task was used to explore participants’ changing physical and emotional states over time and the support they had received in response. Finally, we asked participants about the main things that helped them feel better at each recovery stage and the advice they would give to family and friends on how to best support a child with a similar experience. Interviews were audio‐recorded and transcribed verbatim, removing any identifying information.

### Data analysis

We followed an inductive thematic analysis approach using the six steps outlined by Braun and Clarke (2006, 2021), with analyses conducted in NVivo 12. We additionally incorporated coding reliability elements (i.e., the use of a second coder and discussion with experienced researchers; Guest et al., 2012) to reduce individual bias and ensure data from this vulnerable population were represented as accurately as possible. No data were missing.

One researcher (Katharina Haag) read all transcripts for familiarization before producing initial codes for the entire dataset. Codes were then linked together to create candidate themes. Transcripts were reread to ensure no relevant information had been missed, and, where applicable, codes were added. Particular attention was paid to differences depending on child sex, age, and PTSD status as reported within the respective themes and subthemes. A second coder (Rose McGuire) read 20% of the transcripts and independently developed themes. Researcher agreement was high, and any differences, which mainly concerned thematic structure, were resolved by discussion and data reexamination. Theme and subtheme structure was further revised through feedback from two researchers experienced in child psychopathology and qualitative research (Sarah L. Halligan, Rachel Hiller), although the general thematic content was retained.

## RESULTS

Two main themes were identified that captured participants’ views on (a) factors helping or hindering their recovery and path toward their goal of “returning to normality” and (b) the support received from different sources. For the latter, parents emerged as key contributors, with adolescent peers also playing important roles. There was limited evidence of thematic differences for high versus low levels of PTSS, male versus female sex, or younger versus older age. Any notable differences are discussed.

### Theme 1: Getting back to normal is a major goal, and youths identified a range of barriers and facilitators towards this goal that changed throughout recovery

Getting back to normal was the main long‐term goal for almost all participants. They defined “normal” in various ways and often through concrete criteria, such as having no more physical impairments, being able to resume pretrauma activities, or not being treated differently anymore by family members or peers because of what happened. They also identified different barriers to returning to normality across recovery stages (see subthemes). Of note, one key barrier described by several participants who still showed high PTSS at the time of the interviews was continued exposure to trauma reminders (e.g., ongoing legal procedures, long waits for medical clearance). Furthermore, physical and psychological recovery were often closely linked, with many participants stating that a full physical recovery would lead to them feeling better emotionally. One 10‐year‐old participant (Participant 15) noted, “When I'm back doing everything I could do before—when the teachers are not overcautious, just when everything goes back to normal—is when I'll probably feel better about it than I do now.”

#### Subtheme 1: At the hospital, youth struggle with processing the trauma and complex medical information as well as with their lack of autonomy

In the interviews, participants frequently struggled to recall memories from the immediate aftermath of the PTE but would describe feelings of shock, pain, and anxiety. The participants’ reports indicated that their experience in the ED or hospital was often highly stressful for them and their families, especially when they were given a worrying or potentially life‐changing diagnosis (e.g., the loss of a limb), injuries that initially seemed minor turned out to be life‐threatening (e.g., unnoticed internal bleeds), or the youth participant had to present at several different hospitals to receive a correct diagnosis. A 14‐year old participant (Participant 23) noted, "And they said, 'You have a possible Grade 5 spleen injury rupture.' And then they were like 'You need to be going to [the] hospital to be treated very quickly.' So that was obviously quite traumatic for me and worrying." A small minority of the sample also perceived insensitive treatment at non–child‐specialized EDs, including the dispassionate revealing of a diagnosis and sensationalist behavior regarding an unusual injury, which caused anger and distress and often aggravated the situation. Once admitted, many participants described being scared of having to undergo medical procedures. This was sometimes exacerbated by doctors’ duty to disclose all possible risks, and/or the youths’ own knowledge of potential adverse outcomes. A 14‐year old participant (Participant 20) stated "I remember asking them‐ 'cause I am really scared of anesthetics‐ 'Am I gonna die?' And they were like 'There is a 99% that you will survive, but there's a 1% chance that you'd die.' So that really scared me." Finally, some participants reported being emotionally affected by their parents' reactions, such as seeing them cry for the first time in their lives or perceiving them as being highly distressed, which, in turn, led to increases in some participants' worry or anxiety.

During subsequent hospital stays, sensory stressors, such as pain or noise in mixed‐age wards, general boredom, discomfort, or a lack of visitors could elicit negative moods. Teenagers were particularly affected and frequently advocated for same‐age wards. They also described a loss of autonomy and a lack of privacy resulting from being reliant on others for physical care, which sometimes led to them being short‐tempered and getting into conflicts with their families. Participant 11, who was 15 years old, noted: "What I would have preferred was for [the doctors and nurses] not to make as much of a fuss about it, just let me do it, and go about my way. If I need a blanket, let me go and get a blanket. "Hospitalization far from home tended to aggravate this, as youths and one or both parents often ended up spending large amounts of time together.

Emotional processing of the trauma and its impacts continued during hospitalization. Some individuals initiated conversations with their families, but others were too distressed for discussions. Participants who regained consciousness at this stage of their recovery (i.e., during their hospital stay) following a coma or head injury commonly struggled with suddenly finding themselves at the hospital with no recollection of previous events. This could be intensified if head injuries limited their language and/or cognitive processing abilities. One 13‐year‐old (Participant 3) said:

[I was] just confused just to why I was there. And then when I realized what happened and got told…and started…cause I was quite, like, hazy, and I wasn't sure what was going on, and I couldn't remember stuff.

On the positive side, except for some families with strained relationships, having visitors often improved participants’ well‐being. For example, Participant 25 (age 15 years) described their friends’ visit as a turning point in their recovery: "[Friends visiting for my birthday] changed it, really, made me a lot better. [I] started to eat more, then, and drink… yeah, just this boost of my confidence. [We] just talked about a lot of stuff, and what happened… then they'd got me stuff for my birthday." Being far from home could limit direct social contact, but participants commonly used social media to stay in touch with family and friends.

#### Subtheme 2: Getting home from the hospital is a positive key milestone for youth but often highlights the potential long‐term consequences of the trauma for the first time

For many youths, getting home from the hospital was a point of relief. They described appreciating being back with their families, being in a familiar environment, being able to see their friends again, and gradually regaining their autonomy. This transition commonly helped to rebuild their confidence and positively contributed to their recovery.

One 14‐year‐old (Participant 13) related, “Just being with people that I loved and…knowing that I was safe. So, I was home for quite a few days, and then I felt safe, and back to normal…everyone was just nice to me, and it felt good.”

However, getting home was also when many participants realized how they were different from their pretrauma selves and grasped the potential long‐term impacts of their injury or illness. Some participants tried to accept changes, such as adjustments to their home lives (e.g., needing parents’ help with care, switching rooms to have ground‐floor access) and being unable to rejoin friends for sports or other activities, as temporary necessities; others struggled, especially those who were formerly very active and/or were experiencing more long‐term or permanent changes. These individuals often expressed negative emotions at the restrictions they were experiencing, including anger, sadness, disappointment, and frustration. Fourteen‐year old Participant 23 said: " It properly hit me about two weeks later… as it has been really good weather recently. But is was quite sad for me, because I had to watch lots of my friends go out, do fun stuff. And I was just there, trying to go, but I couldn't." Several participants also described feeling at risk of reexposure to injury or illness, which kept them from resuming their pretrauma activities and interfered with their emotional and physical recovery. A few reported that such worries were exacerbated by receiving poor aftercare following hospital discharge, including not being provided information about follow‐up appointments, not having important medical questions answered, or being unsure whether they were or would soon be fully medically cleared.

Participant 25 (15 years old) noted, “[I am] a bit annoyed, cause [the support] is so good in [the] hospital, but then it's just gone…I just think, ‘I want to ask them some questions.’”

Finally, many participants who reported ongoing PTSS at the time of the interview first started experiencing trauma‐related mental health symptoms once they had returned home. This included early signs of PTSD, anxiety or panic attacks, and/ or depressive symptoms.

#### Subtheme 3: Returning to school is a big step for youth and can have both positive and negative impacts on their recovery

Participants missed, on average, 18 school days posttrauma (range: 2–120). Most spoke in detail about their subsequent return, highlighting its significance in their recovery. Many individuals reported having mixed emotions before returning: They were excited to see their peers and experience relief from the boredom of staying at home, but they also worried about their peers' reactions, their ability to keep up with schoolwork, and physical safety in large crowds. One 14‐year‐old participant noted, “I was happy to be back, ‘cause I really wanted to think about something else. I was obviously nervous about…having to see some people that I hadn't seen yet, I didn't really know how they were gonna react.” Sometimes, when social isolation severely affected a youth participant's well‐being, doctors or parents actively encouraged a return to school for socialization purposes despite the young person's limited capacity to engage in schoolwork.

Upon returning to school, many participants received extensive attention from their peers, including frequent trauma‐related questions. Some enjoyed this, feeling it showed that their peers cared. Participant 9 (12 years) related, "It was quite fun because everyone was like 'why do you have a massive cast on your arm?' So I got to explain it all." Others felt overwhelmed or uncomfortable, especially if this conflicted with their desire for normality. Sometimes, peers had spread rumors or shared confidential information before the participant's return, which caused anger, conflict, and alienation. Most youths reported that over the weeks following their return to school, their peers engaged in warm and supportive behaviors, such as keeping them company or joking around with them. However, some experienced a lack of support, such as exclusion, often due to not being able to join in for physical or break time activities; being made fun of; insensitive reactions; or peers trying to engage them in what may be considered risky behavior given the participant's injuries. One 12‐year‐old participant (Participant 12) said, “So I'm on my own, every break and lunch…well, sometimes [my peers] come, but it's hard for them to remember every time.” Participant 15 (10 years old) noted, “I'd say ‘just don't do headers!’ And my friends are like, ‘Come on, it's not that bad, come on, you won't hurt yourself.’” Participants were often unsure how to address these problems and commonly did not want to involve their teachers. Such difficulties sometimes contributed to continuing trauma‐related distress.

Beyond peer effects, school provisions that promoted child recovery and integration were crucial. Measures taken were commonly driven by participants’ needs and included ensuring the youth participant's physical safety (e.g., allowing them to leave class early to avoid crowded hallways), reducing or offering support with their workload, and allowing chances for quiet time and rest. Some schools made further efforts, providing participants with opportunities to obtain emotional support (i.e., via a school nurse or support teachers) and ensuring they were not excluded by their peers. For example, some participants were allowed to pick friends to join them for a treat, such as an early lunch or playing computer games during break time. Participant 17 (9 years old) said, “I think I found [staying inside during break] helpful, because it made me not so scared that I would get bumped,” and Participant 22 (8 years old) noted, “When it's raining and we can't go outside, I go to Mrs. M.’s office and I play on my Nintendo with other people. I'm allowed to choose people.” Participants highly appreciated such support, which was often linked to higher degrees of perceived well‐being at the time of the interviews.

Some participants described poor communication between the school and their family that hindered their reintegration by making the child or adolescent feel unsure about who knew what. This could make it harder to engage in trauma‐related conversations or ask for support. Although most children and adolescents talked positively about school reintegration, there were a few cases in which participants reported that teachers responded insensitively by not acknowledging what they had been through or reducing their workload to accommodate their needs. One 14‐year‐old participant (Participant 18) said, “The teachers just set homework as usual. I don't think they realized that I still didn't really understand.”

### Theme 2: Social support is varied and can be an important resource for physical and emotional coping

Most participants felt they had received good support after their accident or injury and described far more positive than negative experiences. The extent of support they expected from different sources varied substantially as did the types of support they perceived as helpful at different stages of recovery.

#### Subtheme 1: Parents, and sometimes peers, are the main sources of emotional support, provided in different ways

Despite receiving support from various actors, participants mainly relied on their parents as a key source of emotional support. Although mothers commonly spent more time with the participants, most described both parents as equally important. Outside the family, youths frequently named peers, sometimes specifically sports teams, as their main providers of emotional support. Two adolescent participants had partners but still indicated predominantly relying on their parents for emotional support.

Participants described several types of emotional support, offered by family and close friends, that they perceived as important. Firstly, they described reassurance, which in the immediate trauma aftermath mostly took the form of parents assuring them that everything would be okay. At later recovery stages, family members and friends would, for instance, look up information or tell the participants stories of others persisting through similar challenges to help reassure the youth participant that they could cope. Participant 12 (12 years old) stated, “[My football coach] was saying that I'll get through it, and he knows people that's had the same sorts of thing—that's what my uncle said as well,” and Participant 20 (14 years old) said, “[My friend] was really understanding, he got up pictures of prosthetics and what they look like and how realistic they are. He was like, ‘You're gonna be fine, you have so many friends who support you and everything.’” Similarly, before the young person returned to school, parents often reassured them that they would by physically safe and able to handle their school work and peer attention.

Second, parents often provided guidance and helped their children navigate challenges throughout recovery. At hospital admission, participants reported that parents parsed complex medical information and asked questions to be able to explain important facts to them. One 14‐year old participant said of her father, " He was just trying to understand everything so that when I woke up later, he could explain everything to me. " Later on during recovery, parents provided guidance about adaptive coping and made arrangements with schools to support recovery.

Third, participants appreciated company and availability, especially from peers. They recommended that friends aiming to support youth affected by trauma should regularly check up on them, spend time with them, make themselves available to talk, and make them feel safe, all while not overfocusing on the traumatic event and predominantly treating them normally. Participant 7 (16 years old) offered this insight on posttraumatic support:

Keep checking up on them, make sure they're okay, offer them to come and meet them, see if you can get them anything. ‘Cause, that is what somebody who has been through something like this needs, they just need a friend, someone to talk to, just to be there, just to help cheer them up on a bad day— that's all you really need at the end of the day.

Finally, parents, siblings, and peers tended to engage in protective behaviors, such as ensuring that the child was safe by moderating activities and environments (e.g., setting limits, reducing stimulation). Sometimes, peers or family members also offered help with stressful secondary situations, such as peer problems at school. Such protective behaviors generally made participants feel safe and cared for. One 14‐year‐old participant said, “[My parents] were just very supportive and very kind about the situation. And they did obviously let me know—when I was getting a bit jumpy at times—when to stop, and almost, like, reminded me of my boundaries.”

A few youths, however, reported overprotective reactions. Some participants reported that their parents advised them against engaging in pretrauma activities and encouraged them to keep very safe or that siblings, friends, or teachers treated them extremely cautiously. Such behaviors could lead to frustration and upset, as participants felt they were unnecessary and/or were reminders/ reminded them that they were different from their pretrauma selves. Several participants tried to address this actively with the respective individuals. Participant 20 (14 years old) stated:

It annoyed me a little bit. And I said to my mom, “I don't mind being fussed about, and people treading around me, but it just reminds me that there is something wrong going on.” So I was, like, “Could you just treat me like you did normally?”

Rarely, participants reported that no one in their social environment had done anything to help them feel better or that other people's support strategies were not working. This highlights that there were also limits to the availability and effectiveness of social support.

#### Subtheme 2: Youth appreciate practical support and an acknowledgment of their situation from other individuals but do not expect deep emotional support

Although participants welcomed the company of and sensitive behaviors from siblings, few described them as a main source of emotional support. Most participants simply wanted them to behave normally. Similarly, individuals appreciated visits, gifts, warm support, and reassurance from the wider family or community (e.g., grandparents, neighbors) but rarely spoke to these individuals in depth about their traumatic experiences. Participants generally perceived doctors and nurses at the hospital as doing their jobs well, but they mostly viewed these individuals as points of practical support. Participants expected medical personnel to be kind, attend to their needs, and explain relevant medical information sensitively. A few participants highlighted they would have liked to receive more information from doctors or nurses about the potential emotional consequences of their traumatic experience, which were often not discussed. Finally, as described, individuals appreciated when teachers were supportive of their needs and checked in with them; however, the vast majority of participants did not want to speak to teachers about their trauma in‐depth, if at all, and if they did, it was commonly with school nurses or support teachers.

#### Subtheme 3: Views were mixed on the helpfulness of talking about the traumatic event

Participants differed on whether they discussed the traumatic event with others and if they perceived this to be beneficial. A substantial proportion of participants did not speak to others about the traumatic event frequently, for varying reasons. Some youths, often boys, stated they did not need help with processing the trauma and that they could cope on their own and wanted to focus on the future. Other participants highlighted personality factors, such as being “laid‐back” or not being a “talker.” A desire to avoid being the center of attention was a factor that specifically prevented peer conversations. However, a subgroup of participants avoided speaking or thinking about the traumatic event due to distress and tended to become upset if their family or friends mentioned it. One such youth was Participant 18 (14 years old), who stated, “I'd probably recommend parents to kind of comfort you and not talk about the accident because I didn't really like that…’cause I just couldn't remember it. Yeah, I just didn't like hearing about it.”

Other participants spoke openly about their experiences with parents and peers, with conversations covering both facts and emotional responses; peer discussions were more common among teenage participants. This allowed these individuals to share the mental burden and helped them cope with their initial distress and remaining restrictions. Several participants stated that being able to make light‐hearted jokes about their traumatic experiences with their peers was a marker of positive progress in their emotional recovery. Participant 23 (14 years old) noted, “[My peers and I] have jokes now, and I'm, like, it's funny, so it's fine.” Overall, the frequency of trauma‐related conversations declined as recovery progressed, as relayed by Participant 12 (13 years old), who said, “When it first happened, I talked about it quite a lot, but after a while, I've kind of stopped talking about it, because [I feel better].”

Of note, the subgroup of participants with moderate‐to‐probable PTSD consisted nearly equally of those who still regularly spoke to others about the traumatic event at the time of the interviews as a way of processing the event and its negative consequences and those who avoided the topic altogether due to distress.

## DISCUSSION

The current study aimed to describe children and adolescents’ support needs after trauma exposure and characterize the different support types they received. Practical and/or warm support from various sources was commonplace, but parents and peers were the main providers of emotional support, including with regard to trauma‐related discussions. Participants’ support needs changed over time such that they described appreciating attention and special care while they were at the hospital but valuing increased autonomy and a return to normal life during later stages of recovery. Overall, transitions from the hospital stay to home and from home back to school seem to denote clear and important stages in children's recovery and are important to acknowledge in preventative interventions.

In general, many youths reported receiving helpful support from various sources, which is promising given that low social support has been shown to predict higher levels of PTSS (Trickey et al., 2012). Simply knowing that support was available appeared to be key for most participants, which aligns with findings on the importance of perceived social support among adult trauma survivors (Dunmore et al., 2001). However, youth may vary in their uptake of available support, with some worrying that they could be a burden or not fun to be around. Understanding how such barriers affect received social support is crucial. Of note, both speaking extensively about the trauma and avoiding it appeared to be markers of ongoing distress. Although previous literature has mostly focused on the negative effects of avoidance, the present findings mirror those reported in a recent quantitative study in which the authors identified a group that tended to ruminate or overengage in negative content and, subsequently, reported higher levels of PTSS (Hendrickson et al., 2019). Acknowledging such differences is key to providing tailored interventions.

The present findings concur with ecological models of child posttrauma support, which embed child adjustment within multiple layers of social support (Bronfenbrenner & Morris, 2007; van Wesel et al., 2012). In the present sample, within the closer support network (i.e., parents, families, and peers), parents were described as the main support providers across all ages. This supports previous findings (Alisic et al., 2011a, Hiller et al., 2017) but adds nuance by showing that emotional and practical support, guidance, and opportunities to talk about trauma are particularly important to youth. Adolescents in particular also described peers as key providers of warm or emotional support with whom they talked about their traumatic experiences. Conversely, peer exclusion and a lack of support were commonly associated with worse self‐reported well‐being across all ages, as described in previous studies (van Wesel et al., 2012). Such findings imply that both parents and peers may pose relevant intervention targets for improving children and adolescents’ posttrauma outcomes.

The return to school appeared to be an important point of resocialization. Good parent–school communication and clear school recognition of youths’ needs appear to be important factors for good outcomes during this transition. Although participants commonly wanted teachers to acknowledge that something had happened and treat them appropriately, they did not want to speak about the trauma in detail with their teachers or ask for help if they experienced peer rejection. Previous studies suggest teachers are often unsure about how to best support youth following trauma (Alisic et al., 2012). Our findings indicate that youth do not expect or want teachers to provide psychological support but rather want them to understand and accommodate their current limitations.

Finally, participants’ support needs changed substantially over time. During the hospital stay, when most of the initial trauma processing took place, participants welcomed special attention and warm support from family and friends. However, once they had returned home, their key aim became returning to normality. Simultaneously, individuals often began to realize and be bothered by the long‐term impact of their injury or illness. Successful adjustment may require integrating the exceptional experience of the trauma and its consequences with youths’ pretrauma lives and selves, which has been proposed to happen through a gradual, complex internal negotiation process (Urman et al., 2001). In accordance, participants described appreciating not only reassurance and guidance but also gradually being given more autonomy and being treated as they had been before the incident. Such mixtures of parental encouragement of independence and advising caution against future injury have also been found in real‐life trauma conversations (Mangelsdorf et al., 2019) and may warrant future exploration. Of note, overprotective behaviors served as reminders to the participants that they were not back to normal, consistent with quantitative associations found between parental overprotectiveness and child PTSS (e.g., Williamson et al., 2017).

The current study analyzed a relatively large sample, covering different age groups, trauma types, and stages of recovery. However, some limitations must be discussed. First, although participants otherwise provided balanced accounts, they rarely reported negative parental behaviors. This may be because some parents were present during interviews, although children and adolescents who were interviewed alone did not disclose more information than those whose parents were present. Second, younger children provided somewhat more concrete descriptions, likely due to still‐developing cognitive and language abilities. Same‐aged participants also varied substantially with regard to reflexivity and extraversion, leading to the possible underrepresentation of some perspectives. Furthermore, head injuries affected early support memories in some participants (Hajek et al., 2010). Third, participants were White British only and had more affluent and well‐educated parents than the British average. In addition, participants were recruited from one urban specialist hospital, limiting generalizability. Finally, the findings may not generalize to complex trauma, single‐incident traumatic events without hospitalization, and high‐risk populations.

In sum, we found that youths’ support needs after single‐incident trauma change over time, with a strong reliance on parents, and sometimes peers, for emotional support. Future research should aim to quantitatively replicate such results, with a focus on further differentiating the role of positive versus negative social support and the interactions between social support and other relevant predictors of PTSS.

## OPEN PRACTICES STATEMENT

Transcripts contain sensitive, potentially identifiable information and, consequently, we do not have permission for data sharing.

## AUTHOR NOTE

Katharina Haag is now at the Department of Child Health and Development, Norwegian Institute for Public Health, Oslo, Norway. Rachel Hiller and Rosie McGuire are now at the Division of Psychology and Language Sciences, University College London, London, United Kingdom.

Katharina Haag was funded by a University of Bath PhD Studentship. Additional funding was provided by the Economic and Social Research Council (ES/V002643/1).

The authors wish to thank the children and adolescents and their families who volunteered their time to participate in this study. We would also like to thank Lucie Aplin, Pauline Jackson, Alice Smith, Beth Pittaway, and Sarah Sheedy at Bristol Royal Hospital for Children for their assistance with project recruitment.

## References

[jts22902-bib-0001] Alisic, E. , Boeije, H. R. , Jongmans, M. J. , & Kleber, R. J. (2011a). Children's perspectives on dealing with traumatic events. Journal of Loss and Trauma, 16(6), 477–496. 10.1080/15325024.2011.576979

[jts22902-bib-0002] Alisic, E. , Boeije, H. R. , Jongmans, M. J. , & Kleber, R. J. (2011b). Supporting children after single‐incident trauma: Parents’ views. Clinical Pediatrics, 51(3), 274–282. 10.1177/0009922811423309 21946253

[jts22902-bib-0003] Alisic, E. , Bus, M. , Dulack, W. , Pennings, L. , & Splinter, J. (2012). Teachers' experiences supporting children after traumatic exposure. Journal of Traumatic Stress, 25(1), 98–101. 10.1002/jts.20709 22354512

[jts22902-bib-0004] Alisic, E. , Zalta, A. K. , Van Wesel, F. , Larsen, S. E. , Hafstad, G. S. , Hassanpour, K. , & Smid, G. E. (2014). Rates of post‐traumatic stress disorder in trauma‐exposed children and adolescents: Meta‐analysis. The British Journal of Psychiatry, 204(5), 335–340. 10.1192/bjp.bp.113.131227 24785767

[jts22902-bib-0005] Braun, V. , & Clarke, V. (2006). Using thematic analysis in psychology. Qualitative Research in Psychology, 3(2), 77–101. 10.1191/1478088706qp063oa

[jts22902-bib-0006] Braun, V. , & Clarke, V. (2021). One size fits all? What counts as quality practice in (reflexive) thematic analysis? Qualitative Research in Psychology, 18(3), 328–352. 10.1080/14780887.2020.1769238

[jts22902-bib-0007] Bronfenbrenner, U. , & Morris, P. A. (2007). The bioecological model of human development. In W. Damon & R. M. Lerner (Eds.), Handbook of child psychology (pp. 793–828). Wiley. 10.1002/9780470147658.chpsy0114

[jts22902-bib-0008] Dunmore, E. , Clark, D. M. , & Ehlers, A. (2001). A prospective investigation of the role of cognitive factors in persistent posttraumatic stress disorder (PTSD) after physical or sexual assault. Behaviour Research and Therapy, 39(9), 1063–1084. 10.1016/s0005-7967(00)00088-7 11520012

[jts22902-bib-0009] Guest, G. , MacQueen, K. M. , & Namey, E. E. (2012). Applied thematic analysis. SAGE Publications. 10.4135/9781483384436

[jts22902-bib-0010] Hajek, C. A. , Yeates, K. O. , Gerry Taylor, H. , Bangert, B. , Dietrich, A. , Nuss, K. E. , Rusin, J. , & Wright, M. (2010). Relationships among post‐concussive symptoms and symptoms of PTSD in children following mild traumatic brain injury. Brain Injury, 24(2), 100–109. 10.3109/02699050903508226 20085447 PMC2829882

[jts22902-bib-0011] Hendrickson, M. L. , Abel, M. R. , Vernberg, E. M. , McDonald, K. L. , & Lochman, J. E. (2020). Caregiver–adolescent co‐reminiscing and adolescents’ individual recollections of a devastating tornado: Associations with enduring posttraumatic stress symptoms. Development and Psychopathology, 32(1), 151–161. 10.1017/S0954579418001487 30704541

[jts22902-bib-0012] Hiller, R. M. , Halligan, S. L. , Tomlinson, M. , Stewart, J. , Skeen, S. , & Christie, H. (2017). Post‐trauma coping in the context of significant adversity: A qualitative study of young people living in an urban township in South Africa. BMJ Open, 7(10), e016560. 10.1136/bmjopen-2017-016560 PMC5640056 28988171

[jts22902-bib-0013] Hiller, R. M. , Meiser‐Stedman, R. , Fearon, P. , Lobo, S. , MacKinnon, A. , Fraser, A. , & Halligan, S. L. (2016). Changes in the prevalence and symptom severity of child PTSD in the year following trauma: A meta‐analytic study. Journal of Child Psychology and Psychiatry, 57(8), 884–898. 10.1111/jcpp.12566 27169987 PMC4982080

[jts22902-bib-0014] Hiller, R. M. , Meiser‐Stedman, R. , Lobo, S. , Creswell, C. , Fearon, P. , Ehlers, A. , Murray, L. , & Halligan, S. L. (2018). A longitudinal investigation of the role of parental responses in predicting children's post‐traumatic distress. Journal of Child Psychology and Psychiatry, 59(7), 781–789. 10.1111/jcpp.12846 29197098 PMC6849512

[jts22902-bib-0015] Mangelsdorf, S. N. , Conroy, R. , Mehl, M. R. , Norton, P. J. , & Alisic, E. (2020). Listening to family life after serious pediatric injury: A study of four cases. Family Process, 59(3), 1191–1208. 10.1111/famp.12490 31506948

[jts22902-bib-0016] Marsac, M. L. , Kassam‐Adams, N. , Hildenbrand, A. K. , Nicholls, E. , Winston, F. K. , Leff, S. S. , & Fein, J. (2016). Implementing a trauma‐informed approach in pediatric health care networks. JAMA Pediatrics, 170(1), 70–77. 10.1001/jamapediatrics.2015.2206 26571032 PMC4939592

[jts22902-bib-0017] McGuire, R. , Hiller, R. M. , Cobham, V. , Haag, K. , & Halligan, S. L. (2019). A mixed‐methods investigation of parent‐child posttrauma discussion and the effects of encouraging engagement. European Journal of Psychotraumatology, 10(1), 1644127. 10.1080/20008198.2019.1644127 31489132 PMC6711190

[jts22902-bib-0018] Morley, C. , & Kohrt, B. (2013). Impact of peer support on PTSD, hope, and functional impairment: A mixed‐methods study of child soldiers in Nepal. Journal of Aggression, Maltreatment & Trauma, 22, 714–734. 10.1080/10926771.2013.813882

[jts22902-bib-0019] QSR International. (n.d.). *NVivo* (Version 12) [Computer software]. https://www.qsrinternational.com/nvivo‐qualitative‐data‐analysis‐software/home

[jts22902-bib-0020] Ramsdell, K. D. , Smith, A. J. , Hildenbrand, A. K. , & Marsac, M. L. (2015). Posttraumatic stress in school‐age children and adolescents: medical providers' role from diagnosis to optimal management. Pediatric Health, Medicine and Therapeutics, 6, 167–180. 10.2147/PHMT.S68984 29388603 PMC5683267

[jts22902-bib-0021] Røkholt, E. G. , Schultz, J.‐H. , & Langballe, Å. (2016). Negotiating a new day: Parents’ contributions to supporting students’ school functioning after exposure to trauma. Psychology Research and Behavior Management, 9, 81–93. 10.2147/PRBM.S97229 27175097 PMC4854255

[jts22902-bib-0022] Sachser, C. , Berliner, L. , Holt, T. , Jensen, T. K. , Jungbluth, N. , Risch, E. , Rosner, R. , & Goldbeck, L. (2017). International development and psychometric properties of the Child and Adolescent Trauma Screen (CATS). Journal of Affective Disorders, 210, 189–195. 10.1016/j.jad.2016.12.040 28049104

[jts22902-bib-0023] Salter, E. , & Stallard, P. (2004). Posttraumatic growth in child survivors of a road traffic accident. Journal of Traumatic Stress, 17(4), 335–340. 10.1023/b:Jots.0000038482.53911.01 15462541

[jts22902-bib-0024] Trickey, D. , Siddaway, A. P. , Meiser‐Stedman, R. , Serpell, L. , & Field, A. P. (2012). A meta‐analysis of risk factors for post‐traumatic stress disorder in children and adolescents. Clinical Psychology Review, 32(2), 122–138. 10.1016/j.cpr.2011.12.001 22245560

[jts22902-bib-0025] Urman, M. L. , Funk, J. B. , & Elliott, R. (2001). Children's experiences of traumatic events: The negotiation of normalcy and difference. Clinical Child Psychology and Psychiatry, 6(3), 403–424. 10.1177/1359104501006003009

[jts22902-bib-0026] van Wesel, F. , Boeije, H. , Alisic, E. , & Drost, S. (2012). I'll be working my way back: A qualitative synthesis on the trauma experience of children. Psychological Trauma: Theory, Research, Practice, and Policy, 4(5), 516–526. 10.1037/a0025766

[jts22902-bib-0027] Watson, M. C. , & Errington, G. (2016). Preventing unintentional injuries in children: successful approaches. *Paediatrics and Child Health* , 26(5), 194–199. 10.1016/j.paed.2015.12.006

[jts22902-bib-0028] Williamson, V. , Creswell, C. , Butler, I. , Christie, H. , & Halligan, S. L. (2016). Parental responses to child experiences of trauma following presentation at emergency departments: a qualitative study. BMJ Open, 6(11), e012944. 10.1136/bmjopen-2016-012944 PMC5128846 27821599

[jts22902-bib-0029] Williamson, V. , Creswell, C. , Butler, I. , Christie, H. , & Halligan, S. L. (2019). Parental experiences of supporting children with clinically significant post‐traumatic distress: A qualitative study of families accessing psychological services. Journal of Child & Adolescent Trauma, 12(1), 61–72. 10.1007/s40653-017-0158-8 32318180 PMC7163877

[jts22902-bib-0030] Williamson, V. , Creswell, C. , Fearon, P. , Hiller, R. M. , Walker, J. , & Halligan, S. L. (2017). The role of parenting behaviors in childhood post‐traumatic stress disorder: A meta‐analytic review. Clinical Psychology Review, 53, 1–13. 10.1016/j.cpr.2017.01.005 28137661

